# Prioritizing Zoonoses: A Proposed One Health Tool for Collaborative Decision-Making

**DOI:** 10.1371/journal.pone.0109986

**Published:** 2014-10-10

**Authors:** Cassidy Logan Rist, Carmen Sofia Arriola, Carol Rubin

**Affiliations:** Centers for Disease Control and Prevention, Atlanta, Georgia, United States of America; Kent State University, United States of America

## Abstract

Emerging and re-emerging zoonotic diseases pose a threat to both humans and animals. This common threat is an opportunity for human and animal health agencies to coordinate across sectors in a more effective response to zoonotic diseases. An initial step in the collaborative process is identification of diseases or pathogens of greatest concern so that limited financial and personnel resources can be effectively focused. Unfortunately, in many countries where zoonotic diseases pose the greatest risk, surveillance information that clearly defines burden of disease is not available. We have created a semi-quantitative tool for prioritizing zoonoses in the absence of comprehensive prevalence data. Our tool requires that human and animal health agency representatives jointly identify criteria (e.g., pandemic potential, human morbidity or mortality, economic impact) that are locally appropriate for defining a disease as being of concern. The outcome of this process is a ranked disease list that both human and animal sectors can support for collaborative surveillance, laboratory capacity enhancement, or other identified activities. The tool is described in a five-step process and its utility is demonstrated for the reader.

## Introduction

The majority of emerging or reemerging infectious diseases originate in animals [1], [2], with over 250 zoonoses documented in the literature as newly discovered or rapidly increasing in incidence or geographical range in the past 70 years [3], [4]. In addition to the emergence of zoonotic pathogens, an estimated 20% of all human illness and death in the least developed countries are attributable to endemic zoonoses [5]. Globally, the top 13 zoonoses deemed most impactful to poor livestock keepers in developing countries are responsible for an estimated 2.7 million deaths and 2.4 billion cases of human illness each year; the majority of these diseases also have negative effects on livestock production [4]. The global impact of emerging and endemic zoonoses on both human and animal populations make their control and prevention a natural starting point for collaboration between human and animal health sectors. As collaboration efforts move forward, identifying zoonotic disease priorities of jurisdictional importance to governments and institutions becomes critical.

Given the realities of finite fiscal and personnel resources for both public health and animal health institutions in all countries, joint prioritization of zoonoses has the potential to benefit both sectors as efforts are made to conduct efficient and effective surveillance, develop laboratory capacity, target outbreak response, implement disease control strategies, and identify research activities. However, accomplishing the task of prioritization in a manner that is transparent and useful for all stakeholders can be challenging even in the best of situations; the paucity of quantitative data for decision-making and lack of framework required for multi-sectoral collaboration can significantly impede the process. Taking a collaborative approach to the priority-setting process ensures equal input from stakeholders in both human and animal health sectors, and ideally results in a ranked list of zoonoses that can inform joint efforts in areas of overlapping interest.

Historically recognized methods for prioritization have been adapted by health officials to identify infectious diseases, of both public and animal health importance, for national surveillance and risk-assessment [6]–[12]; several publications have focused specifically on the prioritization of zoonoses [13]–[22]. In general, after determining the pathogens to be prioritized, the ranking processes have employed a hybrid of methods to 1) select the criteria used to define the importance of pathogens, 2) apply weights to individual criteria, and 3) to score the pathogens within each criterion. Criteria weights and associated criteria scores are then combined in some manner to produce the final ranked list of pathogens. The various methods used for criteria selection and weighting, and the scoring of pathogens are often described as qualitative, quantitative, or semi-quantitative in nature based on the scoring system used and the type of data required (Table 1).

**Table 1:** Methods used for criteria selection, weighting and scoring of pathogens.

Method	Definition	Examples
Qualitative	Qualitative methods rely on subjective individualpreference and, in group settings, are often based ona process that creates consensus among group members	Delphi method [28] *;*Subject matter expertopinion
Semi-quantitative	Semi-quantitative methods also rely on individualpreference, but allow choices to be ranked relativeto each other using a numerical scale	Analytic HierarchyProcess [25]; Las Vegasmethod [29]
Quantitative	Quantitative methods rely on numerical scalesthat are designed to reflect objective values(e.g. prevalence or incidence)	Decision Tree Analysis* [30]

*The nature of the questions used in the decision tree will determine if the process is quantitative or semi-quantitative.

Published descriptions of infectious disease prioritization processes vary by the number of pathogens ranked, the number of criteria chosen and the methods used for ranking criteria and scoring pathogens (Table 2). Most recent publications have moved toward using more quantitative methods for prioritization, however all still rely on subject matter expert (SME) opinion at some time during the process. Although a few of the recent publications are focused on prioritization in developing countries [20]–[22], efforts overall remain limited to a small subset of health institutions, particularly to those located in developed countries with greater access to scientific expertise and specific disease prevalence data [13]–[19].

**Table 2:** Summary of publications on the prioritization of infectious diseases at the national or regional level*.

Author	Purpose ofPrioritization(Country/Region)	No. ofPathogensRanked	No. ofCriteriaUsed	Methods Used			
				To SelectCriteria	ToRank/WeightCriteria	To ScorePathogens	To DetermineFinal Pathogen Rank
Doherty J,2000 [7]	To establishpriorities for nationalcommunicabledisease surveillance(Canada)	43	10	Discussionby 10 SMEs	Equal weight	Consensusscoring usingthe Delphimethod [28]by 10 SMEsusing a semi-quantitativescale: 0–5	Sum of pathogenscores
McKenzie Jet al., 2007 [8]	To prioritizepathogens for awildlife diseasesurveillance strategy(New Zealand)	82	3	Not stated	Equal weight	Individualscoring byunstatednumber ofteammembersusing bothquantitativeand semi-quantitativescales. Eachpathogenscored byonly oneperson	Multiplication ofall three criteriascores
Cardoen Set al., 2009 [19]	To prioritize food-and water-bornezoonoses most relevantas hazards in the foodchain (Belgium)	51	5	Not stated	Weightsassigned by 7risk managersusing thesemi-quantitativeLas Vegasmethod [29].Mean scoreused as finalweight	Individualscoring by 35SMEs using asemi-quantitativescale: 0–4.Mean scoreused in finalanalysis	Sum [criterionweight × pathogen score]
Havelaar ATet al., 2010 [17]	To support thedevelopment ofnational surveillancesystems for emergingzoonoses (Netherlands)	86	7	Not stated	Weightsassigned by7 riskmanagers,11 SMEs,and 11medicaland veterinarystudentsusing thequantitativemethod ofprobabilisticinversion [13]	Scored using aquantitativenatural scalewith 4–5 levelsfor eachcriterion. Pointscorerepresentingcentral value inrange used forfinal analysis	Linear model usedto combinecriteria weightswith transformedpoint scores foreach pathogen
Balabanova Yet al., 2011 [10]	To rank commonpathogens based ontheir importance fornational surveillanceand epidemiologicalresearch in order toguide future research(Germany)	127	10	Not stated	Weightsassigned by86 SMEsusing semi-quantitativescoring scale0–10. Averageof medianscore used asfinal weight	Consensus scoringusing the Delphimethod [28] by20 SMEs usingthree-tiered semi-quantitativescoringsystem: −1, 0, 1	Sum [criterionweight × pathogen score]
Humblet MFet al., 2012 [18]	To prioritize 100animal diseases andzoonoses (Europe)	100	57 dividedinto 1 of 5categories	Discussion bySMEs	Weightsassigned by40 SMEsusing thesemi-quantitativeLas Vegasmethod [29]for categoriesand criteria.Mean scoresused as finalweights	Individual scoringby 40 SMEs usingasemi-quantitativescale: 0–7. Meanscore used in finalanalysis	Sum [5 categoryscores] where:Categoryscore = Sum [criterion weight× pathogen score]× category weight
Ng V andSargeant JM,2012 [15]	To describe asystematic andquantitative approachto the prioritizationof zoonoses in NorthAmerica involvingpublic participants(United States andCanada)	62	21	Nominal grouptechnique [31]used in focusgroups with 54participantsfrom medical,veterinary andnon-healthbackgrounds	Criteria scoresdeterminedby emailedsurveys to1,539members ofthe publicusing thequantitativeConjointAnalysismethod [33]	Scored usingquantitative 3–4level scaledefined based onrange of valuesexhibited in theliterature	Hierarchical Bayesmodels fitted toderive CA-weighted scores
Ng V andSargeant JM,2013 [16]	To develop a point-scoring system toderive a recommendedlist of zoonoses forprioritization (UnitedStates and Canada)	62	21	Nominal grouptechnique usedin focus groupswith 54participantsfrom medical,veterinary andnon-healthbackgrounds	Criteria scoresdeterminedby emailedsurveys to1,471 healthprofessionalsusing thequantitativeConjointAnalysismethod [33]	Scored usingquantitative 3–4level scale definedbased on range ofvalues exhibitedin the literature	Hierarchical Bayesmodels fitted toderive CA-weighted scores
Cediel N et al.,2013 [20]	To establish prioritiesfor zoonosessurveillance,prevention and control(Colombia)	32	12	Based oncriteriadevelopedby Krause etal., 2009 [32]	Weightsassigned by12 SMEs usingsemi-quantitativescoring scale 0–12. Average ofmedian scoresused as finalweight	Consensus scoringusing the Delphimethod [28] by12 SMEs usingthree-tieredsemi-quantitativescoring system: −1,0, 1	Sum [criterionweight × pathogen score]
Batzukh Zet al., 2013 [22]	To strengthensurveillance andresponse activitiesand laboratorycapacity betweenhuman, animal andenvironmental sectors(Mongolia)	29	Not stated	Not stated	Not stated	Not stated	Not stated

*Only publications that include a final ranked list of pathogens are referenced in the table.

### One Health Zoonotic Disease Prioritization Tool

In contrast to existing prioritization processes, the One Health Zoonotic Disease Prioritization (OHZDP) Tool was developed specifically to meet the needs of those working in areas where quantitative data on zoonoses are scarce and ties between the human and animal health sectors may be underutilized. Using established qualitative, semi-quantitative and quantitative methods, the OHZDP Tool seeks to build collaboration between diverse stakeholders and provide a dynamic list of prioritized zoonoses that can be used to justify research and allocate funding. Four important requirements of the prioritization tool were identified during the development process. Specifically, the Tool is designed to:

1. Allow equal input from stakeholders in all invested sectors using transparent methods.
2. Accommodate diversity in location (i.e. globally), scale (i.e. local, national, regional), and intended purpose (i.e. project development, surveillance, research activities, etc.) of the prioritization process.
3. Acknowledge data limitations and utilize alternative disease data to create a prioritized list of zoonotic diseases when data specific to the region are not available.
4. Provide outcomes in a timely manner so that participants may give immediate feedback and capitalize on collaborations built during the prioritization process.

## Methods

**Figure 1:**
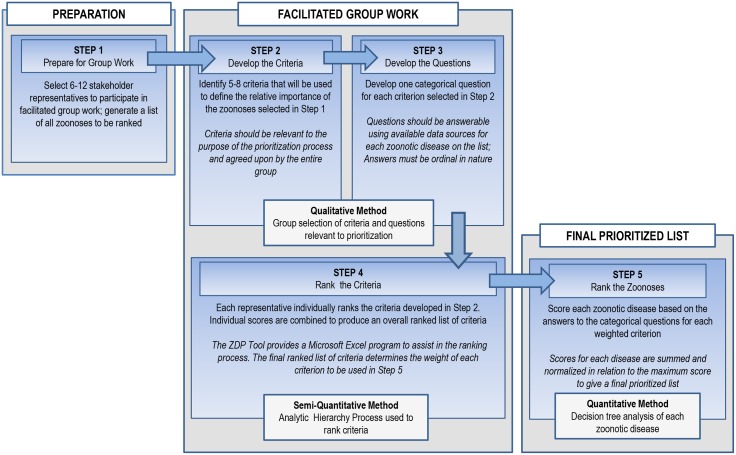
The five steps of the prioritization process using the One Health Zoonotic Disease Prioritization Tool.

The OHZDP Tool addresses the above requirements in a series of five steps (Figure 1). Some of the steps involve group work, while others can be performed by a single individual or subset of the group. The setting in which the group work takes place is assumed to be in-person, as discussion is key to several steps in the process. A moderator familiar with the Tool is optimal to facilitate group discussion and to compile and present results.

In order to provide a clear understanding of the process, an example of the expected outcome is provided after each step in the process is described below. The example is not representative of any specific government or country institution, but is intended to give the reader an idea of how the process proceeds stepwise.

### Step One–Prepare for Group Work

Once the need for joint prioritization is recognized and the intent for the product of the prioritization process is agreed upon (i.e. how the prioritized list will be used for collaboration by the stakeholders), a group of suitable representatives of all stakeholders is identified and asked to participate. Based on focus group research, the recommended number of participants should be between 6 and 12 people in order to balance variation in opinion with a manageable group size [23], [24]; Stakeholder groups should be equally represented in the final group selection. Selected representatives should have a strong working knowledge of their sector’s current zoonotic disease activities and ability to advocate for the use of the final prioritized list in future collaborative efforts.

With the purpose of the prioritization in mind, a list of zoonoses of jurisdictional importance to the stakeholders is generated. This list can be compiled by a single person or group (not necessarily the selected stakeholder representatives), and should make use of all available internal and external sources. The list should be thoughtfully generated and include zoonoses and vector-borne diseases with animal reservoirs suspected to be of local importance; rather than an exhaustive enumeration of all possible zoonotic diseases. The list is brought to the table at the beginning of the group meeting, and may consist of diseases (e.g. Salmonellosis), pathogens (e.g. *Salmonella enteritidis*), and/or groups of pathogens (e.g. food-borne gastrointestinal illness) depending on the level of detail desired. Optimally, the list will include about 15–30 diseases or pathogens.

Example:

- Purpose: To determine which zoonoses will receive funding for joint surveillance projects between a human health agency and an animal health agency in ‘Country X’.
- Stakeholders include both the human and animal health agencies. Each agency chooses five representatives to participate in the prioritization process for a total of 10 participants.
- Prior to the scheduled group work, one representative from both the human and animal health agency work together to develop the list of zoonoses to be ranked. The list includes: all zoonotic pathogens currently under surveillance by either the human or animal health agency; and any zoonoses known to be present in the human or animal population in Country X or in any bordering country as determined by a PubMed literature search, reports to ProMED-mail, the World Health Organization (WHO) and the World Organisation for Animal Health (known as OIE for its acronym in French). The list generated includes 20 zoonoses, all of which are categorized as individual pathogens except for several bacterial foodborne zoonoses (*Escherichia coli, Salmonella spp., and Campylobacter spp.,*), which are included as a single category of “Bacterial Food-Borne Zoonoses”.

### Step Two–Develop the Criteria

The selected group of 6–12 stakeholder representatives meets to brainstorm and develop a list of criteria that will be used to define what qualifies a zoonosis as being important. Five to nine criteria are agreed upon through moderated discussion, but not ranked at this time. The range in number of criteria [5]–[9] has been recognized as optimum for use in the ranking process used in Step 4 of the Tool [25]. The list of criteria is generated by subjective assessment, however the moderator provides examples of criteria used in other published methods for disease prioritization to the group in order to encourage careful consideration of all potentially useful criteria (Table 3).

**Table 3:** Example criteria and categorical questions used in Steps 2 and 3 of the OHZDP Tool to prioritize zoonotic diseases (ZD).

Examples of Criteria for Selection*	Examples of Candidate CategoricalQuestions Used to Define Each Criteria†
Transmission potential betweenhumans and animals	**Q:** Has the ZD caused outbreaks in the country involving animals and humans within the last XX years?
	**A:** Yes or No
Epidemic/pandemic potential in humans	**Q:** Has the ZD caused epidemics in the past XX years?
	**A:** Yes or No
	**Q:** Has the ZD caused pandemics in the past XX years?
	**A:** Yes or No
	**Q:** Has the ZD pathogen been detected in a new location or population (human or animal) in the country in the past XX years?
	**A:** Yes or No
	**Q:** Is the ZD pathogen capable of sustained human-to-human transmission?
	**A1:** Yes or No
	**A2:** Three categories: Never reported, Rare/close contact only, Sustained
Bioterrorism potential	**Q:** Is the ZD listed as select agent (Lists A, B, and C)?
	**A:** Yes or No
Amenability to collaborate/collaborationalready established	**Q:** Do both the Ministry of Health (MoH) and Ministry of Agriculture (MoA) have surveillance/control measures for the ZD?
	**A1:** Yes or No
	**A2:** Three categories: Neither, Either MoH or MoA, Both MoH and MoA
Economic burden of disease	**Q:** What is the ZD case fatality rate in animals without treatment?
	**A:** Four categories: 0–1%, >1–10%, >10–25%, >25%
	**Q:** Is the ZD listed on the OIE list of reportable animal diseases?
	**A:** Yes or No
	**Q:** Does the ZD cause significant (>XX%) decrease in animal productivity?
	**A:** Yes or No
Severity of illness in humans	**Q:** What is the ZD case fatality rate in humans without treatment?
	**A:** Three categories: 0–1%, >1–10%, >10%
	**Q:** Is the ZD case fatality rate >XX% in humans?
	**A:** Yes or No
	**Q:** Can the ZD result in long-term disability?
	**A:** Yes or No
	**Q:** Is case fatality rate greater than XX%, or does the pathogen cause long-term disability in greater than XX% of those infected?
	**A:** Yes or No
Ability to prevent/control the zoonoticdisease in the country	**Q**: Is the ZD listed in country-specific surveillance programs for humans or animals?
	**A1**: Yes or No
	**A2**: Three categories: Neither, human or animal, both human and animal
	**Q:** Is there a known wildlife reservoir for the pathogen?
	**A:** Yes or No
	**Q:** Is there an effective vaccine for the ZD in the primary animal reservoir?
	**A:** Yes or No

*The handout is provided to participants to stimulate conversation and is not intended as an exhaustive list of possibilities.

† Only one categorical question is chosen to represent each criterion.

Example:

- The ten representatives meet and select the following criteria (not listed in any specific order) as important to determining joint surveillance priorities: Bioterrorism potential, severity of illness in humans, economic burden of disease, amenability to collaborate, and epidemic potential.

### Step Three–Develop a Question for Each Criterion

One categorical question is composed for each criterion using the same group of representatives. The questions can have binomial (e.g. yes/no) or multinomial answers that must be ordinal in nature (e.g. <10%, 10–50%, >50–75%, >75%). The ordinal nature of the answers is necessary for the scoring process used in the decision-tree analysis described in Step 5. Numerical cutoff values should be selected carefully, as different cut points will alter scores for some pathogens, and should provide good discrimination among diseases. In order to simplify the process, no more than 5 ordinal categories are recommended; this is consistent with the quantitative scales used by previously described methods [15]–[17].

The Tool provides a list of sample categorical questions for each of the criteria listed (Table 3), however these can and should be modified based on group preference and relevance to the particular purpose of the process. Questions must be structured in such a way that they can be answered by a single person or group using data sources available for all of the pathogens on the initial list. Sources of data to answer the questions are identified or defined at this point, according to the respective question (e.g. CDC website, PubMed literature search, country outbreak or surveillance data, OIE website, WHO website, etc.). Ensuring that questions are answerable provides the qualitative component of the prioritization method and may require unique or innovative thinking during question development, especially in settings where traditional disease data such as prevalence and incidence are lacking.

Example:

The ten representatives develop the following questions and answers to represent criteria selected in Step 2:

- Bioterrorism Potential:

**Q:** Is the pathogen listed as a select agent (Lists A, B, or C)?
**A:** Yes or No
**Source:** As referenced by CDC website.

- Severity of Illness in Humans:

**Q:** Is the case fatality rate for the pathogen/disease in humans greater than 10%?
**A:** Yes or No
**Source:** As referenced by WHO website or published literature specific to the country.

- Economic Burden of Disease:

**Q:** Does the pathogen cause more than 10% mortality in the animal population or more than a 10% decrease in animal productivity?
**A:** Yes or No
**Source:** As referenced by OIE website or published literature specific to the country.

- Epidemic Potential:

**Q:** Has the pathogen been detected in a new location or population (human or animal) within Country X or any bordering country within the past 5 years?
**A:** Yes or No
**Source:** As referenced by Country X outbreak and/or surveillance data, confirmed reports on ProMED mail website, confirmed reports to WHO or OIE.

- Amenability to Collaborate:

**Q:** Do human or animal health laboratories have diagnostic capacity available for the pathogen in Country X?
**A:** Three categories: (Neither) or (At least one- human or animal) **or** (Both human **and** animal labs have capacity)
**Source:** Confirmation from Country X laboratory personnel

### Step Four–Rank the Criteria

The selected criteria are ranked using the semi-quantitative Analytic Hierarchy Process (AHP) [25]. First, each group member individually ranks the criteria using a series of pairwise comparisons of the criteria with a Microsoft Excel program developed as part of the OHZDP Tool to help guide participants through the ranking process. Next, the moderator uses the Excel program to merge responses of all participants, thus creating a ranked list of the criteria determined by the scores provided by each individual. Finally, a sequential (from largest to smallest) weight is assigned for the highest to lowest ranked criterion (e.g. for five selected criteria, the highest ranking criterion is assigned a weight of 5, the second highest a 4, down to the lowest ranking criterion which receives a weight of 1).

Example:

Each of the ten representatives individually ranks the criteria developed in Step 2 using the AHP process with the assistance of the Excel program. The individual scores are combined to produce a final ranked list of criteria, and are given weights based on their rank:

1. Severity of Illness in Humans (weight = 5)
2. Bioterrorism Potential (weight = 4)
3. Economic Burden of Disease (weight = 3)
4. Capacity to Collaborate (weight = 2)
5. Epidemic Potential (weight = 1)

### Step Five–Rank the Zoonotic Diseases

A decision tree is built in Microsoft Excel using the highest ranked criterion as the first node, the second highest ranked criterion as the second node, and so on. The previously formulated categorical questions and answers delineate the path that diseases will follow. The pathogens identified in Step 1 move through the decision tree based on responses to the categorical questions at each node. Responses or “decision branches” are weighted based on the weight given to each criterion in Step 4. When answers are binomial, a score of 1 is applied to one answer and a score of 0 is given to the other answer. The answer given a ‘1’ will receive the full weight of the criterion. For questions with multinomial answers, scores are given in increasing levels determined by dividing the answer’s ordinal position by the total number of answers to the question (e.g. A question with 4 ordinal answers: score for 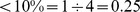; score for 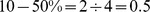; score for 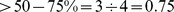; 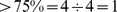). The weight corresponding to the criterion is then multiplied by the answer’s score to get the weighted score for the question (e.g. if the answer to the question was 10–50% and the weight of the criterion was 3, then the weighted score for the question 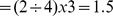). Weighted scores for all questions are summed for each pathogen and normalized in relation to the maximum score to generate the final prioritized list of pathogens. The final product is a list of the original pathogens, presented in a ranked order that is determined by the weighted criteria deemed important by the group.

Example (Figure 2):

**Figure 2:**
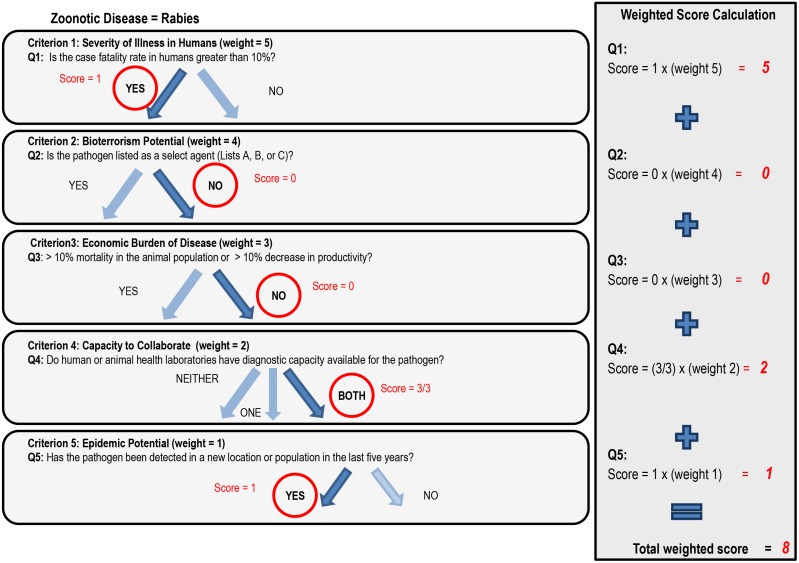
An example of decision tree analysis (Step 5 in the OHZDP Tool) for rabies. The criteria and questions shown are examples only, provided to show the process of how each zoonotic disease is scored. Criteria and questions are developed and given weights by the stakeholder representatives during the facilitated group work in Steps 2–5. Weighted scores for each question are summed to give the total weighted score for each pathogen; total weighted scores are normalized in relation to the maximum pathogen score to give a final ranked list.

- The question for the first criterion (severity of illness in humans) has a binomial answer (yes/no); it was decided that the ‘yes’ answer receives the score of 1. For the example of rabies, the answer to question 1– “Is the case fatality rate in humans greater than 10%?”– is ‘yes,’ therefore the score of 1 is multiplied by the weight of the criterion (weight = 5) and the weighted score = 5. This process is applied to all questions with binomial answers.
- The question for the fourth criterion (capacity to collaborate) has a multinomial answer (neither/at least one/both), with ‘neither’ receiving the lowest score (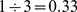), ‘at least one’ receiving the second highest score (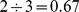) and ‘both’ receiving the highest score (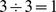). For the example of rabies, the answer to question 4– “Do the human or animal laboratories have diagnostic capacity available for the pathogen in Country X?”–is ‘both’, therefore the score of 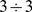 is multiplied by the weight of the criterion (weight = 2), and the weighted score = 2. If the answer was ‘at least one’ then the weighted score would be 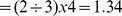.

The final score for rabies is 8, or the sum of the weighted scores for each question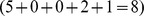. Each of the original 20 zoonoses on the list is scored with this method and the final scores are normalized in relation to the highest score. After all pathogens are scored based on the results of decision tree analysis, the agencies in this fictitious example agree to use available funding to support the top 5 zoonoses on the ranked list to support joint surveillance activities.

## Discussion

As governments and institutions move toward a multi-sectoral approach to zoonotic disease prevention, control, and research, effective channels for collaboration are required. Methods for joint prioritization can provide the means to open communication and build trust as well as provide transparency in the priority decision-making process. The proposed OHZDP Tool provides a process through which a prioritized list of zoonotic pathogens is generated by combining a qualitative method for determining criteria to be used, the semi-quantitative method of the AHP to rank criteria, and the quantitative technique of decision tree analysis to rank the pathogens. The process and methods employed meet the four requirements that were identified during the development phase.

Equal input from all participants is achieved in steps 2 through 4, combining group discussion and individual ranking to generate a weighted set of criteria and associated questions to be used in the decision tree analysis. Although qualitative methods, in this case group agreement on criteria and questions, have been criticized for lack of transparency and for the potential introduction of bias that can occur when employed in group settings [8], the semi-quantitative method of the AHP used to create the combined ranked list of criteria ultimately limits the influence any one person or perspective can have over the decision-making process. However, decisions made based on qualitative and semi-quantitative methods still rely on the selection of participants who may or may not be representative of all stakeholders [26], [27]; thus, the appropriate selection and balance of stakeholders is explicitly emphasized as part of the prioritization process.

The OHZDP Tool provides flexibility to diverse stakeholders invested at local, national, or regional levels by allowing the group to first determine the purpose of the prioritization process and then define criteria and questions relevant for ranking the list of zoonoses. The example of ‘Country X’ used in this paper defines its purpose as ‘funding allocation for disease surveillance at the national level’ and creates a list of all zoonotic pathogens that are geographically relevant and of national interest. Alternatively, the Tool could be used by a university or research institution to determine which zoonotic diseases should be the focus of the upcoming funding cycle. In this case, the list may be smaller and limited to the current investigators’ pathogens of interest or current research.

Strictly quantitative methods provide a more unbiased approach to decision-making because choices are based on data. For example, in health decision-making, people can examine health parameters for different diseases and prioritize them based on burden of disease estimates, provided good quality data are available. Quantitative methods have been applied to prioritize diseases, specifically in developed countries [15]–[17]. However, the methods have rarely been employed in developing countries, where, in general, public health surveillance data are lacking [20]–[22]. The OHZDP Tool uses decision tree analysis in a quantitative manner, relying on the categorical questions to be answerable based on objective data. Because questions are not fixed as they are for most other described methods [7], [10], [13], [15]–[20], this allows participants to make use of disease data they know to be available for the scope and purpose of the prioritization process.

Integral components of collaborative work include respect for time and the ability to act on decisions made by the group. Although the OHZDP Tool was designed with the desired outcome of a prioritized list in mind, it also builds collaboration through the process. By coming together as a group, representatives are able to understand how other stakeholders view the importance of zoonoses to their relative sectors; developing criteria together helps to frame the zoonoses in relation to group priorities. The OHZDP Tool provides Microsoft Excel programs to assist in group ranking of criteria (Step 2) and in the final ranking of pathogens in the decision tree process (Step 5), which together with facilitated group work results in a rapid and transparent method for zoonoses prioritization.

In the pilot trials of the tool, Steps 2–4 could be completed in a one-day time period. The decision tree process, which can be completed primarily by the facilitator or other assigned individuals, can take up to another half or full day of time depending on the number of zoonoses selected for analysis. This means that participants know the results of the ranked criteria at the end of the first group session, and the results for the final ranked pathogen list can be ready within 24 hours. Rapid turnaround allows further discussion and a timely output that can be used immediately for its intended purpose.

To provide further clarification for the five steps in the OHZDP Tool, the complete output from one pilot study is provided in the supporting information. For this particular pilot study, the authors brought together six professionals currently active in zoonotic disease research. Three of the participants were asked to assume the role of representatives from a fictional country’s Ministry of Health and three from the Ministry of Agriculture. They were provided a list of 17 zoonoses (Table S1) and assisted through Steps 2–4 of the OHZDP Tool (Tables S2, S3). Step 5 was completed by the authors (Table S4, S5), and the final prioritized list is presented in Table S6.

The authors are aware that further validation of the OHZDP Tool is an optimal next step; however, the OHZDP Tool is similar to other tools in its design, as each step of the prioritization process (selection of criteria, weighting of criteria, scoring of pathogens, and final determination of pathogen rank), employs a previously validated prioritization method (Table 2). Quantitative comparison with the tools that already exist may be difficult as the majority require at least basic surveillance data [7], [10], [15]–[19]. The OHZDP Tool is designed to be used in countries where surveillance data are lacking and even expert opinion may not be sufficient to accurately estimate zoonotic disease prevalence or incidence in human and/or animal populations.

Future pilots of the Tool will assess its internal validity by repeating the process using different representatives from the same stakeholder groups and comparing the final prioritized lists using non-parametric tests. In addition, results of sensitivity tests for the impact of each criterion and the importance of assigned criteria weights will be documented to assess their influence on the final ranking of the diseases. And finally, although the range of number of participants, criteria and categories for questions were provided using documented sources [23]–[25], the robustness of the Tool can be further evaluated by altering these values and comparing results. A Facilitator Manual for the OHZDP Tool, including instructions for the Excel programs is available from the authors upon request for those interested in participating in ongoing evaluation and validation of the Tool.

In summary, the OHZDP Tool was developed for use by organizations or institutions interested in prioritizing zoonoses; the purpose of the prioritization can vary based on stakeholder needs, but can ultimately serve to identify areas of overlapping interest, focus the use of limited resources, and maximize the impact of zoonotic disease related activities. The Tool presented here differs from others in its ability to combine individual and group decision making processes together with limited disease data in a manner that is flexible enough to meet the needs of multi-sectoral groups with differing levels of jurisdictional reach. The authors feel the tool offers a transparent and timely process for those who wish to prioritize zoonoses using a collaborative approach and welcome any questions or comments on the Tool’s potential utility.

## Supporting Information

Table S1 **Step 1: List of zoonotic diseases compiled for pilot testing of the OHZDP Tool.**
(DOCX)

Table S2 **Steps 2 and 3: Criteria and associated questions selected by the pilot group.**
(DOCX)

Table S3 **Step 4: Excel program for group ranking of criteria using the Analytic Hierarchy Process.**
(XLSX)

Table S4 **Step 5: Answers to the questions for each of the 17 selected zoonoses.**
(DOCX)

Table S5 **Step 5: Excel program used to rank the pathogens using decision tree analysis.**
(XLSX)

Table S6 **Step 5: Prioritized list of the 17 zoonoses based on their final, normalized scores in the decision-tree analysis.**
(DOCX)
