# Supplementary material for: Prioritizing Zoonoses: A Proposed One Health Tool for Collaborative Decision-Making
Source: PLoS One. 2014 Oct 10;9(10):e109986. doi: 10.1371/journal.pone.0109986 (PMC4193859; doi:10.1371/journal.pone.0109986)
Supplement: Table S1 — Step 1: List of zoonotic diseases compiled for pilot testing of the OHZDP Tool. (DOCX) [file pone.0109986.s001.docx]

| **Table S1**. Step 1: List of zoonotic diseases compiled for pilot testing of the OHZDP Tool^*^ |
| --- |
| Anthrax |
| Bovine Tuberculosis |
| Brucellosis |
| Cysticercosis |
| Enteric Pathogens (Salmonella, E. coli, Campylobacter, Giardia, Cryptosporidium) |
| Hantavirus |
| Hepatitis E virus |
| Japanese Encephalitis virus |
| Leptospirosis |
| Lyme disease |
| Melioidosis |
| Nipah virus |
| Plague |
| Q Fever |
| Rabies |
| Tick-borne encephalitis virus |
| West Nile virus |
| *Listed in alphabetical order |
