# Supplementary material for: Prioritizing Zoonoses: A Proposed One Health Tool for Collaborative Decision-Making
Source: PLoS One. 2014 Oct 10;9(10):e109986. doi: 10.1371/journal.pone.0109986 (PMC4193859; doi:10.1371/journal.pone.0109986)
Supplement: Table S2 — Steps 2 and 3: Criteria and associated questions selected by the pilot group. (DOCX) [file pone.0109986.s002.docx]

| **Table S2.** Steps 2 and 3: Criteria and associated questions selected by the pilot group. For each criterion the group developed a question, answers, and provided the sources where the answers to the questions would be drawn from. Additionally, the group determined how the answers would be weighted (e.g. Does the “Yes” or “No” answer receive the full weight of the criterion?). | | | |
| --- | --- | --- | --- |
| **Criterion** | **Question** | **Answer (categorical weight)** | **Source for Answers** |
| Human Morbidity/Mortality | Is case fatality rate greater than 5% if untreated, or does the pathogen cause long term disability in greater than 10% of people infected? | a) Yes (1)  b) No (0) | - CDC  - WHO |
| Food Security | In the absence of a control program, does the pathogen/disease cause greater than 5% mortality or decrease production more than 25% in food producing animals? | a) Yes (1)  b) No (0) | - FAO  - OIE  - Center for Food Security and Public Health- Iowa State |
| Ability to Prevent/Control | Is there an effective vaccine in humans or animals or an effective treatment in humans? | a) No (1/3)  b) Human or Animal (2/3)  c) Human AND Animal (3/3) | - CDC  - WHO  - OIE  - Center for Food Security and Public Health- Iowa State |
| Amenability to Collaborate | Surveillance/control measures in the Ministry of Health (MOH) and/or the Ministry of Agriculture (MOA)? | a) No (1/3)  b) MOH **or** MOA (2/3)  c) MOH **and** MOA (3/3) | - Inquiry to ‘Country X’ |
| Bioterrorism | Is the pathogen listed as class A or B on the select agent list? | a) Yes (1)  b) No (0) | - CDC |
