# Supplementary material for: Prioritizing Zoonoses: A Proposed One Health Tool for Collaborative Decision-Making
Source: PLoS One. 2014 Oct 10;9(10):e109986. doi: 10.1371/journal.pone.0109986 (PMC4193859; doi:10.1371/journal.pone.0109986)
Supplement: Table S4 — Step 5: Answers to the questions for each of the 17 selected zoonoses. (DOCX) [file pone.0109986.s004.docx]

| **Table S4.**  Step 5: Answers to the five questions selected to represent the five criteria for each of the 17 selected zoonoses (See Table S2) | | | | | |
| --- | --- | --- | --- | --- | --- |
|  | **Human**  **Morbidity**  **And Mortality** | **Food Security** | **Ability to**  **Control/Prevent** | **Amenability**  **To Collaborate** | **Bioterrorism** |
|  | Yes/No | Yes/No | No/OR/AND | No/OR/AND | Yes/No |
| Anthrax | Yes | Yes | AND | OR (animal) | Yes |
| Bovine Tuberculosis | Yes | No | AND | No | No |
| Brucellosis | No | Yes | AND | AND | Yes |
| Cysticercosis | Yes | No | OR (human) | No | No |
| Enteric Pathogens | No | No | OR (humans) | OR (human) | Yes |
| Hantavirus | Yes | No | No | No | Yes |
| Hepatitis E virus | No | No | No | OR (human) | No |
| Japanese Encephalitis virus | Yes | Yes | AND | AND | No |
| Leptospirosis | Yes | Yes | AND | AND | No |
| Lyme disease | Yes | No | OR^1^ (humans) | No | No |
| Melioidosis | Yes | No | OR (humans) | No | Yes |
| Nipah virus | Yes | Yes | No | No | No |
| Plague | Yes | No | OR (humans) | AND | Yes |
| Q Fever | No | Yes | AND^2^ | No | Yes |
| Rabies | Yes | No | AND | AND | No |
| Tick-borne encephalitis virus | Yes | No | No | No | No |
| West Nile virus | Yes | No | OR (animal) | OR (animal) | No |
| ^1^ Although a vaccine exists for canines, the vaccine is not used in the natural wildlife reservoirs of Lyme Disease  ^2^ Vaccines are not available for domesticated ruminants in the U.S., however they are available in some countries | | | | | |
