# Supplementary material for: Prioritizing Zoonoses: A Proposed One Health Tool for Collaborative Decision-Making
Source: PLoS One. 2014 Oct 10;9(10):e109986. doi: 10.1371/journal.pone.0109986 (PMC4193859; doi:10.1371/journal.pone.0109986)
Supplement: Table S6 — Step 5: Prioritized list of the 17 zoonoses based on their final, normalized scores in the decision-tree analysis. (DOCX) [file pone.0109986.s006.docx]

| **Table S6.** Step 5: Prioritized list of the 17 zoonoses based on their final, normalized scores in the decision-tree analysis | |
| --- | --- |
| Zoonosis | Score |
| Anthrax | 1.000 |
| Leptospirosis, Japanese Encephalitis virus | 0.977 |
| Rabies | 0.767 |
| Plague | 0.744 |
| Brucellosis, Nipah virus | 0.698 |
| Bovine Tuberculosis | 0.674 |
| Melioidosis | 0.651 |
| West Nile virus | 0.628 |
| Q Fever | 0.605 |
| Cysticercosis, Lyme disease | 0.581 |
| Hantavirus | 0.558 |
| Tick-borne encephalitis virus | 0.488 |
| Enteric Pathogens | 0.349 |
| Hepatitis E virus | 0.186 |
